# Child safety programs for primary school children decrease the injury severity of dog bites

**DOI:** 10.1007/s00431-021-04256-z

**Published:** 2021-09-17

**Authors:** Bernhard Kienesberger, Christoph Arneitz, Vanessa Wolfschluckner, Christina Flucher, Peter Spitzer, Georg Singer, Christoph Castellani, Holger Till, Johannes Schalamon

**Affiliations:** 1 Department of Pediatric and Adolescent Surgery, Medical University of Graz, Auenbruggerplatz 34, 8036 Graz, Austria; 2 Research Center for Childhood Accidents, Safe Kids Austria, Graz, Austria

**Keywords:** Dog bites, Dog injuries, Injury prevention, Pediatric dog injuries

## Abstract

This study focuses on the impact of a prevention program regarding dog bites in children. As a consequence of our previous investigation in 2005, we have initiated a child safety program for primary school children starting January 2008 until present to teach children how to avoid dog attacks and how to behave in case of an attack. In our retrospective study, we analyzed all patients younger than 15 years presenting with dog-related injuries between 2014 and 2018. As the main indicator for success of the prevention measures taken, we have defined the severity of injury in comparison to our previous study. Out of 296 children with dog-related injuries, 212 (71.6%) had sustained a dog bite. In the vast majority (*n* = 195; 92%), these patients presented with minor injuries; the extremities were most commonly affected (*n* = 100; 47%). Injuries to the head (*n* = 95; 45%) and trunk (*n* = 18; 8%) were less frequent. The proportion of severe injuries (8%) was significantly lower compared to our previous study, where 26% of children presented with severe injuries necessitating surgical intervention, while the number of patients requiring in-hospital treatment declined from 27.5% in the period 1994–2003 to 9.0% in the period between 2014 and 2018 (*p* < 0.05).

*Conclusion*: Teaching of primary school children may effectively reduce the injury severity of dog bites.

**Table:** 

**What is Known:** *• Dog bites are a substantial healthcare problem especially in children.*	
**What is New:** *• This study shows that a broad-based prevention program for primary school children can effectively decrease the severity but not the frequency of dog bite injuries in children.*

**What is Known:**

*• Dog bites are a substantial healthcare problem especially in children.*

**What is New:**

*• This study shows that a broad-based prevention program for primary school children can effectively decrease the severity but not the frequency of dog bite injuries in children.*

## Introduction

While dogs benefit children in many ways, the number of dog bites is a substantial health care problem and remains constantly high. For example, over 4 million dog bites are reported in the USA every year; 2–3/100,000 people need hospital admission and the annual incidence of bites in children aged under 15 years is 22/1,000 [1–3]. The majority of the victims are children and in some areas, the numbers of dog bites are rising despite a large number of publications on this topic [4].

Although most dog bites occur at home [5] and in up to 80% of the bites, the dogs are familiar to the children [6, 7]; governmental prevention measures focus on public incidents and regulations concerning leash and muzzle. In order to successfully prevent incidents with dogs, parents or caretakers should concentrate on understanding and influencing the dog’s conspicuous behavioral patterns and not just on safety equipment [8]. Moreover, the mandatory reporting of dog bite incidents to gain epidemiologic data is not required by law in many European countries [9] and as a consequence, there is a lack of long-term observational studies regarding child safety programs and prevention of dog bites.

An accurate understanding of where, why, and how dog bites occur and what associated factors may affect the outcome is of pivotal importance for any prevention efforts. In 2005, we have published the incidence, mechanism, and circumstances of dog bites in pediatric patients [6]. As a consequence, local interventions to increase public awareness were established in collaboration with the Austrian Committee for Injury Prevention in Childhood (Safe Kids Austria; https://grosse-schuetzen-kleine.at) in order to lower the rate and severity of dog bite injuries in our catchment area. The aim of our study was to analyze the efficacy of our targeted prevention program after a 10-year period.

## Patients and methods

### Prevention program

The independent ethics committee (institutional review board) approved this study (EK: 31–135 ex 18/19). As a consequence of our previous study [6], we have initiated a child safety program starting from January 2008 until the present to teach children how to avoid dog bite incidents and how to behave in case of an attack. The target group consisted of primary school-aged children during the first three years at school (6–9 years of age). Every year about 2,400 children are enrolled in primary schools in Graz, Austria [10]. About 80% (1,800–2,000 children annually) were reached by our prevention program. A code of behavior while handling a dog was part of the visiting program at the child safety house, an institution where parents and children are informed and trained to cope correctly with daily potential hazards (https://grosse-schuetzen-kleine.at/baerenburg; annual number of approximately 1,500 pediatric visitors). A special dog training with veterinarians and/or dog trainers was completed by approximately 400 children per year.

The prevention program promoted precautionary behavior when approaching a dog and advice for handling a dog safely and confidently. It included more than 100 child-dog workshops under supervision of dog trainers and/or veterinarians and the production and distribution of educational and information materials suitable for children (which had a print run of 20,000 copies) at primary schools. The workshops consisted of a 45-min training including a demonstration of various “rules of behavior” around dogs, e.g., how to approach and pet a dog, how to read the dog’s mood, and how to recognize a friendly, angry, or frightened dog. Children were instructed to ask the dog owner for permission, avoid eye contact, not to disturb the dog while sleeping or eating, to move slowly and quietly near dogs, and to let the dog sniff the hand before touching. In case of an attack, a protective body posture was demonstrated.

In addition, we have addressed child-dog interactions at the annual child safety days (following several articles in the local press) and during our guided tours of the child safety house (“Bärenburg”), where specific safety equipment is presented and workshops focusing on incident prevention are held. The program has continued until present, while the primary attendants became adolescents and influenced children and other children with their behavior and knowledge. Therefore, we investigated dog bites in children younger than 15 years of age.

### Data analysis

The Department of Pediatric Surgery is the only level-I-trauma referral center for children in Graz, Austria. For the present study and building on our previous publication [6], data of all children younger than 15 years of age seeking medical attention at our Department after dog-related injuries between 2014 and 2018 were analyzed. Bites were studied retrospectively following our initial protocol [6] including the severity of injuries and breed-related data. Since 2010 there is a compulsory register for dog breeds in Austria, the quality and availability of data on local breed distribution improved. To exclude a possible breed-related bias of injury severity compared to the prior survey, the current numbers of local breeds were reinvestigated.

As the main indicator for success of the prevention measures taken, we have defined the severity of injury in comparison to our previous study. Deep and penetrating wounds requiring surgical closure or repeated interventions were defined as severe injuries (categories II and III, respectively). Superficial lesions, scratches, and smaller lacerations (when a firm wound closure without a suture was possible) were considered minor injuries (category I).

The breed-related risk index was calculated by dividing the representation of a dog breed among the total dog population by the representation of this breed among all evaluated dog bites.

### Statistical analysis

For comparison of categorical data like the percentage of patients requiring in-hospital treatment or the proportion of severe injuries in the two periods (1994–2003 vs. 2014–2018), the *χ*^2^ test was used to determine the statistical significance. Statistical comparison of the mean number of dog bites per year between the two periods was performed with the Mann–Whitney *U*-test. Age comparison of the different localizations of dog bite injuries was performed with the Kruskal–Wallis test followed by pairwise testing by Mann–Whitney *U*-test with a Bonferroni correction applied. All computations were performed using the statistical software package SPSS 22 for Windows (SPSS, Inc., Chicago, IL). *p* values of < 0.05 were considered to be statistically significant.

## Results

### Dog-related injuries

**Fig. 1:**
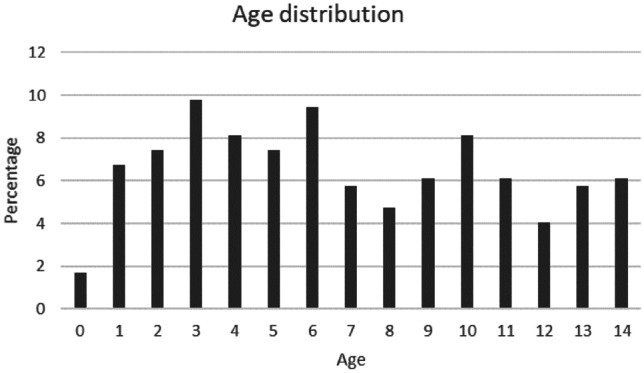
Age distribution of 296 children with dog-related injuries. The mean age of the patients was 6.7 years

**Fig. 2:**
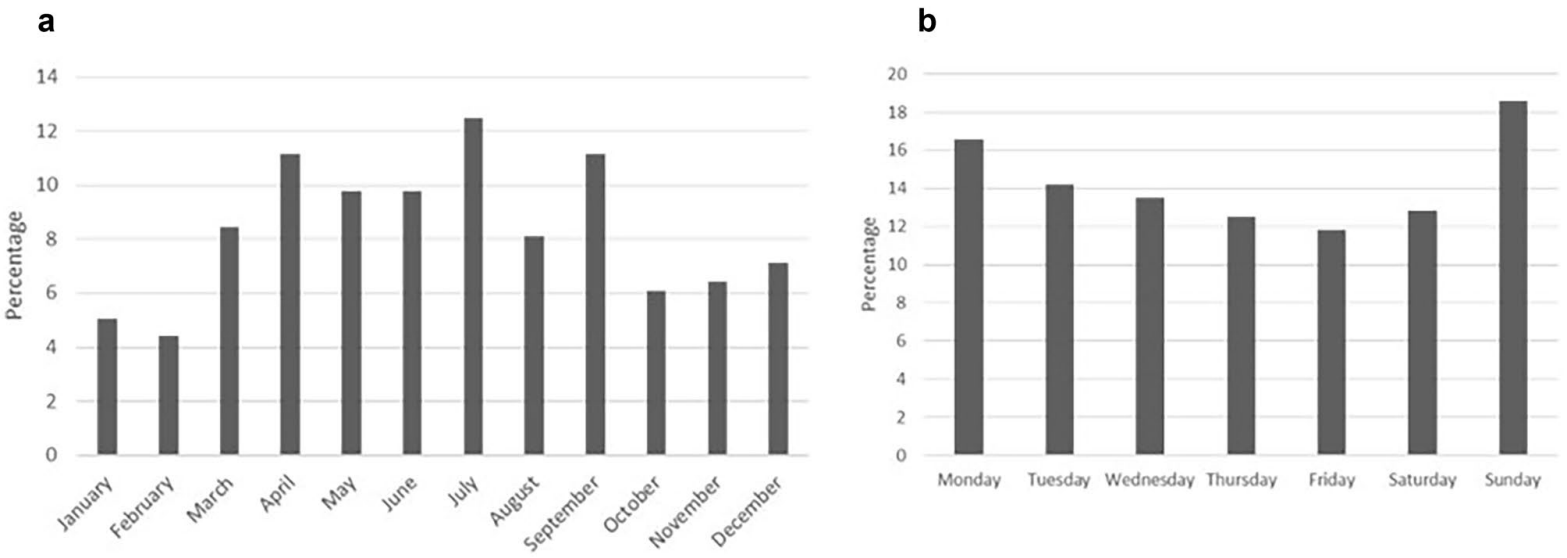
Distribution of 296 dog-related injuries per month (**a**) and per weekday (**b**). Note the peak in July and on Sundays

There were 296 children younger than 15 years presenting with dog-related injuries in our study. Three-quarters of those incidents (*n* = 222; 75%) occurred in the age group 0–10 years (Fig. 1). The gender was equally distributed (m: *n* = 148; f: *n* = 148) and the majority of the victims were injured during the summer with a peak in July (*n* = 38; 13%) and on Sundays (*n* = 56; 19%) as shown in Fig. 2.

Out of these 296 dog-related injuries, 212 (72%) presented with dog bites (mean age of the patients 6.5 years; standard deviation (SD) 3.8 years) while 28% of the treated injuries (*n* = 84; mean age 7.1 years; SD 2.6 years) resulted from incidents that occurred with or near the dog, but were not dog bites. Among these “non-bite” cases, the most frequent ones were falls due to underestimation of the dog’s force when pulling the leash or jumping at the child while playing (*n* = 43; 51%) followed by injuries related to the dog’s leash (e.g., the dog pulls on the leash causing a finger injury; *n* = 28; 33%), falls over the dog (*n* = 11; 13%), and others (*n* = 2; 3%).

### Dog bites

Patients suffering from dog bites (*n* = 212 out of 296; 72%) mainly presented with minor injuries (category I; *n* = 195; 92%) and the extremities were most commonly affected (*n* = 100; 47%; upper extremities *n* = 43, lower extremities *n* = 57). Injuries to the head (*n* = 95; 45%) and trunk (*n* = 17; 8%) were less frequent. However, children with injuries to the head were significantly younger (mean age 4.6 years; SD 3.2) compared to children with injuries to the upper extremities (mean age 7.6 years; SD 3.9; *p* < 0.05) and to the lower extremities (mean age 9.1 years; SD 3.3; *p* < 0.05). We noted no significant difference between patients with injuries to the head compared to patients with injuries to the trunk (mean age 6.8 years, SD 3.4; *p* = 0.123). Inpatient treatment was required in 19 children (9%), the majority of these had severe injuries (category II and III) requiring operative intervention or repeated surgical treatment. The proportion of severe injuries (*n* = 17 out of 212; 8%) was significantly lower compared to our previous study, where 26% (*n* = 89 out of 341) children presented with severe injuries necessitating surgical intervention (*p* = 0.033). However, the mean number of dog bites per year did not decrease (1994–2003: mean number per year 37.8 vs. 2014–2018: mean number per year 42.4, *p* = 0.267). Additionally, the percentage of patients requiring in-hospital treatment has declined from 27.5% in 2005 to 9.0% in 2019 (*p* = 0.041).

### Characteristics of the biting dogs

In 183 of 212 cases (86%), the exact breed of the dog that injured a child could be determined. The breed-related proportion of two-thirds of the attacking dogs is shown in Table 1. While a standard height of cross-breed dogs is not available, all attacking dogs with distinct known breeds in our recent study were classified as large (> 44-cm acromial height) according to local veterinary guidelines. The only exception was the Dachshund (acromial height of approximately 23 cm), which accounted for 2.7% (*n* = 5) of the dog bites.

**Table 1:** Local distribution of breeds and their related dog bites comparing the results of the present study to our previous report (6) including the associated Risk Index

**Dog breed**	**Dog population, 2004 (6)**	**Dog bites, 1994–2003 (6)**	**Dog population, 2019**	**Dog bites, 2014–2018**	**Risk index**
Cross-breed	28.0%	13% (*n* = 39)	26.5%	27.3% (*n* = 50)	1.0
Labrador/retriever	8.2%	4% (*n* = 11)	7.1%	13.1% (*n* = 24)	1.8
German shepherd	12.0%	34% (*n* = 105)	2.7%	15.8% (*n* = 29)	5.8
Terrier	8.1%	5% (*n* = 15)	2.6%	4.3% (*n* = 8)	1.7
Dachshund	5.2%	7% (*n* = 22)	2.4%	2.7% (*n* = 5)	1.1
Dobermann	1.1%	3% (*n* = 8)	1.5%	4.4% (*n* = 8)	2.9
Bernese dog	1.7%	1% (*n* = 3)	1.4%	3.8% (*n* = 7)	2.7
Rottweiler	1.1%	1% (*n* = 3)	1.3%	2.7 (*n* = 5)	2.0

The data about the distribution of the dog population was collected from the local community dog register. The risk index was calculated by dividing the representation of a dog breed among the total dog population by the representation of this breed among all evaluated dog bites

In the majority of the cases (69%; *n* = 146), the dogs were familiar to the children (being injured by their own dog in 23%; *n* = 49), while in 31% (*n* = 66) of the cases, the attacking dogs were considered unfamiliar.

## Discussion

The present study confirms our previous results as well as the results of other authors [5, 11] regarding the circumstances of incidents: In most cases, the attacking dogs were familiar to the children and the most common location of dog attacks was the home setting where the use of leash and muzzle is uncommon. Both of our investigations are consistent with the results of Khan and co-workers who have described that the majority of registered injuries are due to bite incidents by larger breeds [12]. The authors presumed that bites of smaller breeds less frequently result in severe injuries requiring medical attention.

As our main result, the proportion of severe injuries in the present study was significantly lower compared to our previous investigation, while the absolute numbers of children seeking medical attention showed a mild upward trend; the annual average numbers of dog bite patients increased from 34 in the period from 1994 to 2003 to 42.4 in the period from 2014 to 2018. We hypothesize that our prevention program was responsible for both: While our teaching may have had a positive impact on the most dangerous situations in the coexistence of children and dogs, our campaign may have raised public awareness resulting in hospital visits even in case of minor injuries. In addition, children may feel more comfortable being around dogs, which may lead to an increase in bites observed.

Children are at a high risk of injury during an unprovoked encounter with a stray dog outdoors. Since such attacks are rare, prevention is difficult and should be addressed to dog owners. However, the majority of attacks occur at home while feeding, petting, and playing; parental supervision is present in almost half of the cases [11]. Several predictors of bites have previously been identified: The breed group, incident location, the age of the victim, dog sex and age, and the attitude and knowledge of the victim as well as the training of the dog [7, 13]. While most of these factors are hard to influence, Chapman et al. could show that a simple 30-min lesson for children aged 7 to 8 years “increased appreciably the precautionary behavior of young children around strange dogs in the short term” [14]. The American Academy of Pediatrics recommends close supervision of small children when a dog is near, no matter if it is a family dog [15]. Other authors promote training of dogs as the most effective prevention strategy [16]. These strategies may be applicable for infants and preschool children but will not apply to older children since constant surveillance is difficult in children older than six years of age due to their more courageous and self-confident behavior while certain dog breeds may not respond to training to the same extent. Education in school-aged children on injury prevention has proven its effectiveness before in prevention of drowning and brain injuries [17, 18]. Our study indicates that teaching of primary school-aged children may successfully reduce the severity of dog attacks within the whole population and even in smaller children, since the educated children act as multipliers among families and friends. Furthermore, a modification of behavior may prevent dog bites of both familiar and strange dogs.

Limitations of the present study include that the changing trend in local breed distribution and the breed-related risk index may have influenced the number and severity of dog bites as well as changes in dog training efforts by local dog owners. However, breed distribution was similar despite a period of more than a decade and large dogs were still most frequently attacking. Moreover, we do not know whether the children presenting with dog bite injuries took part in our prevention program. The authors would like to emphasize the fact that any dog is capable of biting.

## Conclusion

Teaching of primary school children may effectively reduce the severity of dog bite injuries. Therefore, public authorities should increase awareness and support on this topic and roll out a nationwide program in order to effectively prevent dog bite injuries in children.

## Data Availability

Excel data sheets are available on request.

## Abbreviations

SD: Standard deviation
*p*: *p*-Value

## Code availability

N/A
